# COVID-related confinement experience in people with major neurocognitive disorders and their caregivers in new aquitaine region, France

**DOI:** 10.1192/j.eurpsy.2021.113

**Published:** 2021-08-13

**Authors:** B. Calvet

**Affiliations:** Old Age Psychiatry, Esquirol hospital center, Limoges, France

**Keywords:** Neurocognitive disorders, Covid, sanitary confinement, BPSD

## Abstract

The COVID-19 epidemic is an unexpected global event that has shaken up the organisation of care in France. The spread of the epidemic was limited thanks to the confinement of people from Tuesday 17 March 2020 to Monday 11 May 2020. However, this confinement led to a change in the care of vulnerable people, including people suffering from neurocognitive disorders (NCDs). The aim of this study is to question people suffering from NCDs and their family carers about their experiences during the period of confinement introduced in connection with the COVID-19 epidemic and on any physical and/or functional consequences.

**Methods:**

All persons whose memory consultations at CMRR Limoges were cancelled during the period of confinement (17 March to 11 May 2020) were contacted by telephone by the nurses or psychologists at CMRR.

**Results:**

The experience of the confinement episode as well as the deconfinement are studied. The survey records the clinical changes in patients and the medical/medical-social events that occurred during this period. The impact of the aids maintained and the place where people live is studied.

**Discussion:**

Confinement is an exceptional measure that makes it possible to reduce the risk of contagion in the event of an epidemic, at the risk of harmful consequences for people weakened by a NCDs. In the event of an epidemic episode in the future, this study could help to define the arrangements to be put in place to better protect people suffering from NCDs and their family caregivers.

**Disclosure:**

No significant relationships.

